# Patterns of social inequalities across pregnancy and birth outcomes: a comparison of individual and neighborhood socioeconomic measures

**DOI:** 10.1186/s12884-014-0393-z

**Published:** 2015-03-23

**Authors:** Nihaya Daoud, Patricia O’Campo, Anita Minh, Marcelo L Urquia, Susie Dzakpasu, Maureen Heaman, Janusz Kaczorowski, Cheryl Levitt, Janet Smylie, Beverley Chalmers

**Affiliations:** Centre for Research on Inner City Health, Li Ka Shing Knowledge Institute, St. Michael Hospital, 209 Victoria Street, third floor, Toronto, ON M5C 1 N8 Canada; Department of Public Health, Faculty of Health Sciences, Ben-Gurion University of the Negev, P.O. Box 653, Beer Sheva, 84015 Israel; Dalla Lana School of Public Health, University of Toronto, Toronto, ON Canada; Maternal and Infant Health Section, Surveillance and Analysis Division, Public Health Agency of Canada, Ottawa, ON Canada; College of Nursing, Faculty of Health Sciences, University of Manitoba, Winnipeg, MB Canada; Département de médecine de famille et de médecine d’urgence and CRCHUM, Université de Montréal, Hôtel-Dieu Hospital, Montréal, Québec Canada; Department of Family Medicine, Faculty of Health Sciences, McMaster University, Hamilton, ON Canada; Well Living House Action Research Centre for Indigenous Infant, Child and Family Health and Wellbeing, Saint Michael’s Hospital, Toronto, ON Canada; Department of Obstetrics and Gynaecology, Ottawa Hospital Research Unit, University of Ottawa, Ottawa, Canada

**Keywords:** Socioeconomic inequalities, Social patterning, Maternal and child health (MCH), Individual and neighborhood socioeconomic measures, Pregnancy and birth

## Abstract

**Background:**

This paper identifies patterns of health inequalities (consistency and magnitude) of socioeconomic disparities for multiple maternal and child health (MCH) outcomes that represent different health care needs of mothers and infants.

**Methods:**

Using cross-sectional national data (unweighted sample = 6,421, weighted =76,508) from the Canadian Maternity Experiences Survey linked with 2006 Canadian census data, we categorized 25 health indicators of mothers of singletons into five groups of MCH outcomes (A. maternal and infant health status indicators; B. prenatal care; C. maternal experience of labor and delivery; D. neonatal medical care; and E. postpartum infant care and maternal perceptions of health care services). We then examined the association of these health indicators with individual socioeconomic position (SEP) (education and income), neighborhood SEP and combined SEP (a four-level measure of low and high individual and neighborhood SEP), and compared the magnitude (odds ratios and 95% confidence intervals) and direction of the associations within and between MCH outcome groups.

**Results:**

We observed consistent positive gradients of socioeconomic inequalities within most groups and for 23/25 MCH outcomes. However, more significant associations and stronger gradients were observed for the MCH outcomes in the maternal and infant health status group as opposed to other groups. The neonatal medical care outcomes were weakly associated with SEP. The direction of associations was negative between some SEP measures and HIV testing, timing of the first ultrasound, caesarean section, epidural for vaginal births, infant needing non-routine neonatal care after discharge and any breastfeeding at 3 or 6 months.

Gradients were steep for individual SEP but moderate for neighborhood SEP. Combined SEP had no consistent gradients but the subcategory of low individual-high neighborhood SEP often showed the poorest health outcomes compared to the categories within this SEP grouping.

**Conclusion:**

By examining SEP gradients in multiple MCH outcomes categorized into groups of health care needs, we identified large and consistent inequalities both within and between these groups. Our results suggest differences in pathways and mechanisms contributing to SEP inequalities across groups of MCH outcomes that can be examined in future research and inform prioritization of policies for reducing these inequalities.

**Electronic supplementary material:**

The online version of this article (doi:10.1186/s12884-014-0393-z) contains supplementary material, which is available to authorized users.

## Background

The World Health Organization defines health inequalities as “differences in health status or the distribution of health determinants between different population groups” [1]. Socioeconomic inequalities in health refer to health gradients related to socioeconomic position (SEP) [2], where those in the lower social strata have poorer health than those in the higher ends of the social hierarchy [3-5]. Social patterning of socioeconomic inequalities in health point to root causes, pathways, and mechanisms [6]. that manifest in society as social class and power relations [7,8]. Measuring the social patterns of socioeconomic inequalities in health can guide the focus on SEP measures for research and help policy makers prioritize specific areas regarding health and health care needs [7,9].

Maternal SEP is known to be a strong correlate of numerous maternal and child health (MCH) outcomes. Low individual SEP (e.g. education and income) has been associated with adverse pregnancy and birth outcomes [10-15], and delayed prenatal care [16,17]. Different SEP measures capture unique aspects and pathways of relative or absolute socioeconomic advantage that can relate differently to MCH indicators [18]. For example, income can help mothers purchase items to meet basic needs, such as food, medication, and transportation to maternal and child health care services, while maternal education reflects not just the individual and household economic dimension of SEP, but also access to information and knowledge, problem-solving skills, social networks and involvement, and social prestige, all of which might be important for MCH [4]. Education reflects life-course SEP [19], including parents’ SEP during childhood and adolescence, access to higher education, work opportunities, and income during adulthood. Previous studies on birth outcomes, maternal behaviors, and prenatal care have shown moderate correlations between education and income SEP measures [10,20]. In addition, a systematic review of studies in industrialized countries showed that income is less frequently associated with birth outcomes than education [18]. However, this might be different in low-income countries where income is critical for MCH [21]. Thus education captures different aspects of SEP compared to what is captured by household income. Looked at together, information on SEP inequalities for each of these SEP measures can help generate hypotheses for future research on pathways and areas of intervention.

Beyond individual SEP measures, more recent studies suggest that neighborhood poverty is an independent risk factor for lower infant birth weight and poorer birth outcomes [22,23], higher maternal smoking during pregnancy [24], and lower utilization of prenatal care [25]. Neighborhood SEP, which reflects larger societal processes impacting the economic and social development of residential areas [26], is gaining more attention in health research. To date, a small but growing set of studies on MCH outcomes have included neighborhood SEP measures [4]. Neighborhood SEP can relate to MCH outcomes via access to and availability of physical and psychosocial residential resources during childbearing years. These factors are determined by larger policies and not by individual-level achievements. Most studies have found small effects of neighborhood SEP measures on health [13,23,27-29].

Recent studies have increasingly begun including measures to capture the impact of different levels of SEP (individual and contextual) [30-32]. Inclusion of measures at multiple levels would unearth the consequences of the deeply rooted processes that shape and determine health and health inequalities [33,34]. Typically studies focus on only one or a few MCH outcomes to study the health inequalities around pregnancy and birth. We argue that measuring SEP inequalities across groups of MCH outcomes can yield information that will help policy makers prioritize areas for reducing these inequalities.

In this paper, drawing on social ecological approaches of explaining SEP inequalities [26], we argue that a better understanding of the social inequalities in health depends on moving beyond the typical examination of a single health outcome and use of one SEP measure [9]. Rather, a systematic overview of the magnitude and direction of SEP inequalities across groups of MCH outcomes around pregnancy and birth simultaneously while using different SEP measures in a large national data set can: 1. help assess the consistency and direction of the SEP gradient and prioritize MCH needs and focused areas with larger inequalities around pregnancy and birth; [9] 2. determine if inequalities are arising from different levels (individual and neighborhood) of SEP processes; and 3. combining results from the two first activities, can help to generate hypotheses for future research on the pathways and mechanisms leading to particular social patterns of SEP inequalities, and suggest strategies for reducing these inequalities. Using different levels of SEP measures is pertinent for measuring health inequalities [4,20,27]. Individual and neighborhood SEP measures reflect different attributes and factors that can jointly or independently impact health and health outcomes [22,27].

In the current study, we sought to provide a comprehensive overview of MCH inequalities by exploring the consistency, direction, and magnitude of inequalities within and between groups of MCH outcomes across individual- and neighborhood-level SEP measures using a large national sample. We categorized 25 health indicators into five MCH groups that represent different areas of interest and different health care needs of the women and children before pregnancy, during pregnancy, and after birth. These include: A. maternal and infant health status indicators; B. prenatal care; C. labor and delivery experiences; D. Medical care during neonatal period; and E. postpartum infant care and maternal reflections on overall experiences of health care. We then examined the consistency and direction of the associations of these MCH outcomes by different SEP measures. Separating outcomes into groups—differentiating health status from service or procedural outcomes—was important as we anticipated, in the context of universal access to health care, that SEP gradients would be small or non-existent for service or procedural outcomes while, stronger SEP gradients would be observed for health status outcomes, which may depend on access to the means of ensuring healthier lifestyles (e.g., secure and rewarding jobs, quality housing, good nutrition). Exploring these inequalities within and between the different groups of MCH outcomes across different SEP measures might help in generating hypotheses about the pathways to these inequalities and facilitate policies to reduce the inequalities.

## Methods

### Data

We linked data from the Canadian Maternity Experiences Survey (MES), a national, complex and unweighted sample of 6,421 (weighted sample =76,508) women sampled from the 2006 Canadian census, to neighborhood data from the same census [35]. Detailed information about the MES can be found elsewhere [36,37], but, briefly, this survey was conducted in 2006 to collect data concerning pregnancy and birth in a national sample of women [35,37]. Women were selected from the 2006 census using stratified random sampling without replacement. A total of 8,244 women met the eligibility criteria of being 15 years of age or older, having had a singleton live birth, and living with their baby at the time of data collection [37]. The response rate was 78%. After applying survey weights, the 6,421 respondents represent 76,508 women in the population [36]. Data were collected through a computer-assisted telephone interview and lasted 45 minutes. Infants were between 5 and 10 months of age at the time of the interview.

The census data were reported at the level of dissemination areas (DAs), a census unit of 400 to 700 persons [38]. Only one woman in each DA was interviewed for the MES [5,39].

The MES project was presented to Health Canada’s Science Advisory Board, Health Canada’s Research Ethics Board and the Federal Privacy Commissioner, and was approved by Statistics Canada’s Policy Committee [36]. Approvals for this analysis were obtained from the St. Michael’s Hospital Research Ethics Board and the Research Data Centre Access Granting Committee of Statistics Canada.

### Measures

The demographic variables (age and parity) and SEP measures are detailed in Table 1.

**Table 1:** **Definitions of socio-demographic and socioeconomic variables and their distribution**

**Variables & definitions**	**Categories**	**Total sample (weighted)**	
		N*	**%**
***Socio-demographic variables***			
**Age**	Total	**68705**	**100**
Maternal age at birth of baby	<20 years	1563	2.27
	20 to 24	8383	12.20
	25 to 29	23120	33.65
	30 to 34	23448	34.13
	35 to 39	10171	14.80
	40 to 50	2020	2.94
**Parity**	Total	**68705**	**100**
Past births and/or still-births	Yes	30209	43.97
***Independent Variables: Socioeconomic Position (SEP) measures***			
**Individual SEP measures**			
**Education**	Total	**68705**	**100**
Highest level of maternal education	Less than high school (HS)	4632	6.74
	HS diploma	12898	18.77
	More than HS (ref.)	51175	74.49
**Household income (LICO)**	Total	**68705**	**100**
At/below or above the low-income cutoff point (LICO) [40]	Below or at LICO	13710	19.96
	Above LICO (ref)	54995	80.04
**Neighborhood-level SEP measure**			
**Percent of households below LICO in the neighborhood**	Total	**68705**	**100.00**
Place of residence located within a census dissemination area with a composition of 15% or more households below LICO	High SEP: <= 15% of households below or at LICO (ref)	52118	75.86
Data source: Census 2006 all other variables are from the MES	Low SEP: >15% of households below LICO	16587	24.14
**Combined SEP measure**			
**Two-level categorical SEP variable, Individual and neighborhood**	Total	**68705**	**100**
Combined variable: a four-level categorical variable combining respondents’ household income with %LICO in a neighborhood	Low Individual SEP-	6240	9.08
	low neighbouhood SEP		
	Low Individual SEP-high neighborhood SEP	7470	10.87
	High Individual SEP-low neighborhood SEP	10347	15.06
	High Individual SEP-High neighborhood SEP (ref)	44648	64.98

*Due to missing data, the total number for some variables does not sum up to 68705.

The SEP measures included:

- *Individual-level SEP,* included highest level of education received by the mother and household income measured by the low income cut-off point (LICO). LICO reflects whether the respondent lives in a household that spends 20 percentage points more of their after-tax income on food, shelter and clothing than the average family of a similar size, thus leaving less income for other expenses, such as health, education, transportation and recreation [40]. We considered LICO an individual-level SEP measure.
- *Neighborhood-level SEP*, is a contextual variable we created by linking the neighborhood DA LICO we obtained from the 2006 Canadian census data to each woman’s survey data. We calculated the proportion of neighborhoods with below LICO households and divided it into two categories: high-SEP neighborhoods (15% or fewer households are below LICO) and low-SEP neighborhoods (more than 15% of households are below LICO) [39].
- *Combined SEP,* was measured by a 4-level categorical variable combining respondents’ household income with the percent of households below the LICO in the neighborhood. The SEP categories in this variable were: 1. low individual-low neighborhood; 2. low individual-high neighborhood; 3. high individual-low neighborhood; and 4. high individual-high neighborhood.

Table 2 presents detailed definitions of the MCH outcomes, including reference categories. Reference groups were chosen based on those most commonly reported in the literature or, in some cases, based on the category where there was the largest sample. As mentioned above, we organized the 25 MCH outcomes we used in the study into five groups based on areas of interest and periods around pregnancy and birth. Each of the five groups of MCH outcomes represents different health care needs of the mother and/or her infant during pregnancy and after birth. The groups of MCH outcomes were as follows:

A. *Maternal and infant health status indicators:* This group represented an overview of the health conditions of a mother and an infant. It included maternal health outcomes after birth (self-rated health and postpartum depression), pre-pregnancy body mass index (BMI), and infant birth outcomes (preterm birth and small for gestational age);
B. *Prenatal care:* This group included adherence to health care recommendations for women during pregnancy. It was measured by time of first prenatal care visit, time of ultrasound, HIV testing, receiving enough information during pregnancy and weight gained during pregnancy;
C. *Labor and delivery experiences:* This group included health care services provided for the women during delivery and birth. It was measured by type of birth, epidural, shaving, and position for vaginal birth.
D. *Medical care during the neonatal period:* This group represented the health care provided for the infant during the first week after birth. It was measured by the following variables: infant hospital readmission, infant needing non-routine care after discharge, admission to Neonatal Intensive Care Unit (NICU), and greater than average length of hospital stay after caesarean or vaginal birth; and
E. *Postpartum infant care and maternal reflection on overall experience of labor and birth*: This group of variables represented infant care provided by a mother after birth and maternal assessment of the overall care she experienced during her pregnancy and after birth. It included four variables: any breastfeeding at 3 months, any breastfeeding at 6 months, satisfaction with various aspects of maternity care, and satisfaction with overall experience of pregnancy and birth.

**Table 2:** **Definition of maternal and child health (MCH) outcome variables and the distribution these variables in the Canadian maternity experiences survey**

**Variables & definitions**	**Categories** ^**ɸ**^	Total sample (weighted)*	
		**N**	**%**
***A Maternal and infant health status indicators***			
**Preterm Birth**			
Infant was born before 37 weeks gestation	Total	**66611**	**100.00**
	Yes	4125	6.19
	No (ref.)	62487	93.81
**Small for Gestational Age**			
Baby’s birth weight below the 10^th^ percentile for his/her gestational age and sex	Total	**66461**	**100.00**
	Yes	5277	7.94
	No (ref.)	61184	92.06
**Self rated health**	Total	**68675**	**100.00**
Mother’s rating of her overall health (postpartum)	Fair/poor	3455	5.03
	Good/very good (ref.)	65220	94.97
**Postpartum Depression**	Total	**68160**	**100.00**
Maternal score on the Edinburgh Postpartum Depression scale	> = 13 on the EPDS	4942	7.25
	less than 13 on the EPDS (ref.)	63219	92.75
**Pre-pregnancy BMI**	Total	**67668**	**100.00**
(Body mass index)	Under 18.5	3780	5.59
	Normal BMI (18.5 – 24.9) (ref.)	40333	59.60
	Over 24.9	23556	34.81
***B Prenatal care***			
**First prenatal care visit timing**	Total	**68010**	**100.00**
	14 weeks or more	3346	4.92
	Before 14 weeks (ref.)	64664	95.08
**Timing of ultrasound**			
Timing of first ultrasound during pregnancy	Total	**66833**	**100.00**
	18 weeks or more	22443	33.58
	Before 18 weeks (ref.)	44390	66.42
**HIV testing**	Total	**68705**	**100.00**
HIV testing of mothers who had prenatal care visits (94.9% of mothers)	Not tested	11010	16.03
	Don’t know	6457	9.40
	Yes had the test (ref.)	51238	74.58
**Received enough information during pregnancy**	Total	**68688**	**100.00**
An index based on 6 yes/no questions on information received on physical, emotional changes, warning signs, tests, and medications and medical test/procedures that may be required during pregnancy; the index was calculated by creating a count variable	None	5144	7.49
	Received Info (ref.)	63544	92.51
**Weight gained during pregnancy (kg)**	Total	**67261**	**100.00**
Mothers’ weight gain during pregnancy based on the Canadian Gestational Weight Gain recommendations	Below recommended range	12585	18.71
	Within recommended range (ref.)	21871	32.52
	Above recommended range	32805	48.77
***C Labor and delivery experiences***			
**Type of birth**	Total	**68705**	**100.00**
	Caesarean	18105	26.35
	Vaginal (ref.)	50600	73.65
**Epidural for a vaginal birth (not for caesarean section)**	Total	**50533**	**100.00**
Of respondents who underwent a vaginal birth, indicates whether respondent used an epidural or spinal anesthetic during labor/birth of the baby.	Yes	27203	53.83
	No (ref.)	23331	46.17
**Position for vaginal birth**	Total	**50518**	**100.00**
Which of the following best describes your position when (baby’s name) was born? 1. Lying on your side, 2. Propped up or sitting 3. Lying flat on your back, 4. Some other position.	Lying flat	23589	46.69
	Not lying flat (ref.)	26928	53.31
**Shaving for a vaginal birth, not caesarean sections**	Total	**50301**	**100.00**
	Yes (ref.)	7576	15.06
	No	42725	84.94
***D Medical care during the neonatal period***			
**Infant hospital readmission**			
Whether the baby has stayed at hospital overnight since he/she was born.	Total	**68659**	**100.00**
	Yes	5161	7.52
	No (ref.)	63498	92.48
**Infant needing non-routine neonatal care after discharge**	Total	**68667**	**100.00**
Whether the baby had a non-routine check-up since the time of birth.	Yes	33845	49.29
	No (ref.)	34822	50.71
**Baby admitted to NICU after birth**	Total	**68632**	**100.00**
Indicates if baby was admitted to NICU immediately after birth.	Yes	8703	12.68
	No (ref.)	59930	87.32
**Length of hospital stay after birth**	**Vaginal Birth** (Total)	**49552**	**100.00**
	More than average (3–4 days)	12216	24.65
	2 days (ref.)	37335	75.35
	**Caesarean** (Total)	**18105**	**100.00**
	More than average (3–4 days)	2983	16.47
	2 days (ref.)	15122	83.53
***E Postpartum infant care and maternal reflections on the overall experience of health care***			
**Any breastfeeding at 3 months**	Total	68705	100.00
Whether respondent was breastfeeding at 3 months.	Stopped by 3 months	36116	52.57
	Yes (ref.)	32589	47.43
**Any breastfeeding at 6 months**	Total	68705	100**.00**
Whether respondent was breastfeeding at 6 months.	Stopped by 6 months	40493	58.94
	Yes (ref.)	28212	41.06
**Overall experience with labor and birth “**Overall, would you describe the experience of labor and birth as: 1. Very negative, 2 Somewhat negative, 3. Neither negative nor positive, 4. Somewhat positive, 5. Very positive”	Total	**68422**	**100.00**
	Neutral or dissatisfied: Categories 1–3 (very negative, somewhat negative and neither negative nor positive)	13441	19.64
	Satisfied (ref.): Categories 4–5 (somewhat positive and very positive)	54981	80.36
**Satisfaction with various aspects of maternity care** “Think back to your entire pregnancy, labor and birth, and immediate postpartum experience, overall how satisfied or dissatisfied were you with: 1.information given, 2. respect, 3. compassion shown, 4. competency, 5.concern for privacy, 6. respondent involvement in decision making.”	Total	**68674**	**100.00**
	Neutral or dissatisfied: Categories 1–3 (very negative, somewhat negative and neither negative nor positive)	12824	18.67
Categories: 1. Very negative, 2 Somewhat negative,	Satisfied (ref.): categories 4–5 (somewhat positive and very positive).	55850	81.33
3. Neither negative nor positive, 4. Somewhat positive, 5. Very positive			

^**ɸ**^Reference groups were chosen as those most commonly reported in the literature or in some cases for the category where there was the largest sample.

*Due to missing data, the total number for some variables does not sum up to 68705.

### Data analysis

We followed the MES Users’ Guide [41] for applying the weights [36]. Missing data were less than 10% for most of the variables. Bivariate associations between each SEP measure and MCH outcomes were calculated using estimates of the proportions and odds ratios (ORs) with 95% confidence intervals (95% CI). We then estimated ORs using multivariable logistic regression models for each of the MCH outcomes while taking into consideration potential confounders of age and parity (the condition or fact of having given birth previously). The models were as follows: Model 1, adjusted for one SEP measure only; Model 2, adjusted for one SEP measure and age; Model 3, adjusted for one SEP measure and parity; and Model 4, was adjusted for on SEP measure, age and parity. We determined that a variable (age or parity) was a confounder when the SEP estimates results changed by 10% or more from the unadjusted model (bivariate associations). However, this change was not noticed and therefore we decided to present only bivariate results in this paper. We assessed the patterns of inequalities based on the direction and magnitude of each association, while comparing the ORs of poor health category with the better health category (reference category) for each SEP measure. The level of significance was set to 10%.

## Results

The distribution of the study demographic and socioeconomic variables is shown in Table 1.

Most women (94.7%) were between 20 and 40 years of age, and about 44% were multiparous. The majority of women had more than a high school diploma (74.5%), a household income above the LICO (80%), and lived in high-SEP neighborhoods (75.9%). The combined SEP showed that about 65% of the women had high individual-high neighborhood SEP, about 9.1% had low individual-low neighborhood SEP, almost 10.9% had low individual-high neighborhood SEP and the rest (15.06%) had high individual-low neighborhood SEP.

Table 2 shows the distribution of the MCH outcomes in five groups of variables.

**Figure 1:**
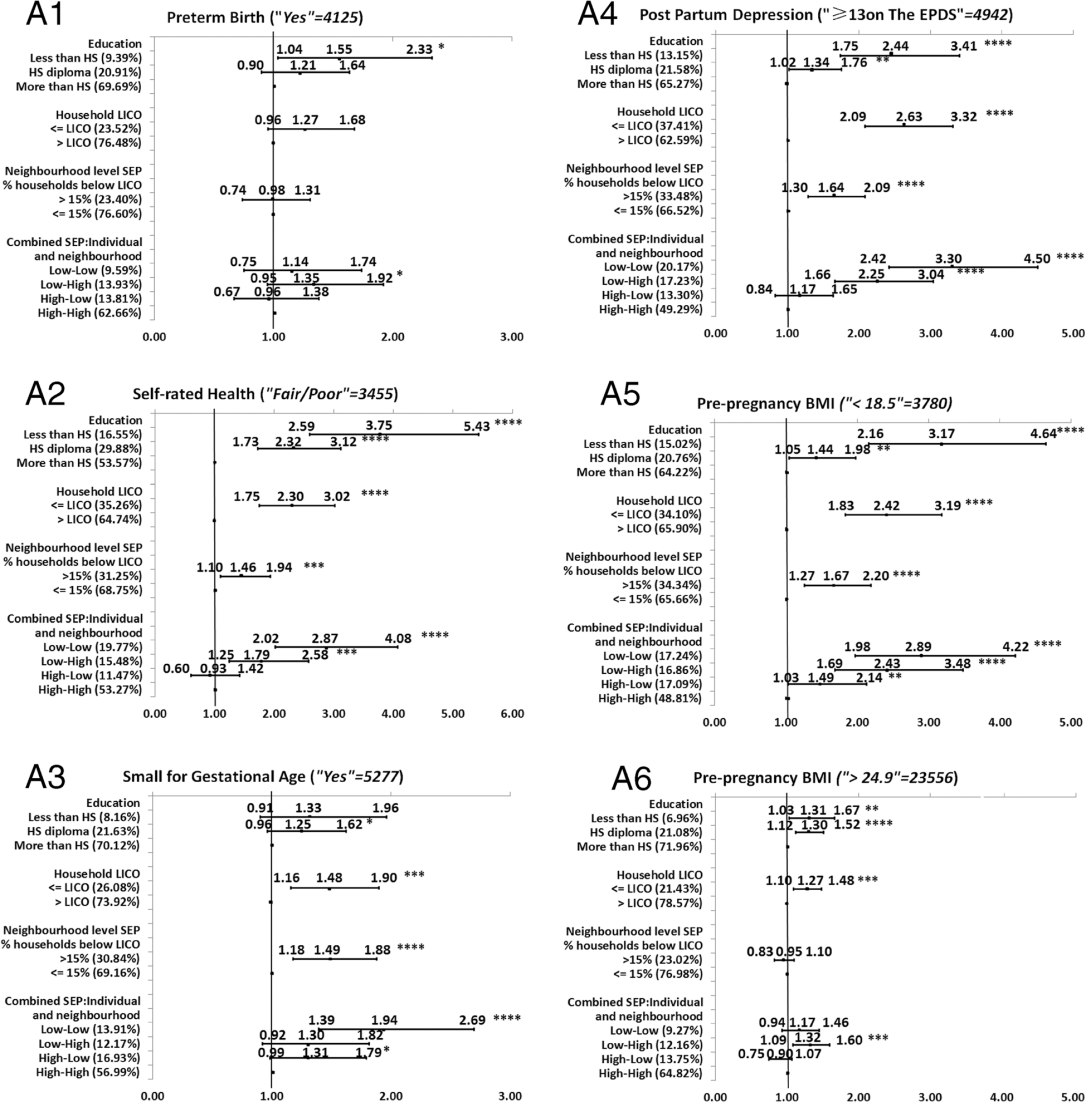
**Associations between individual level SEP (education and household income), neighborhood SEP and combined SEP measures, and the group of maternal and child health status indicators.**

**Figure 2:**
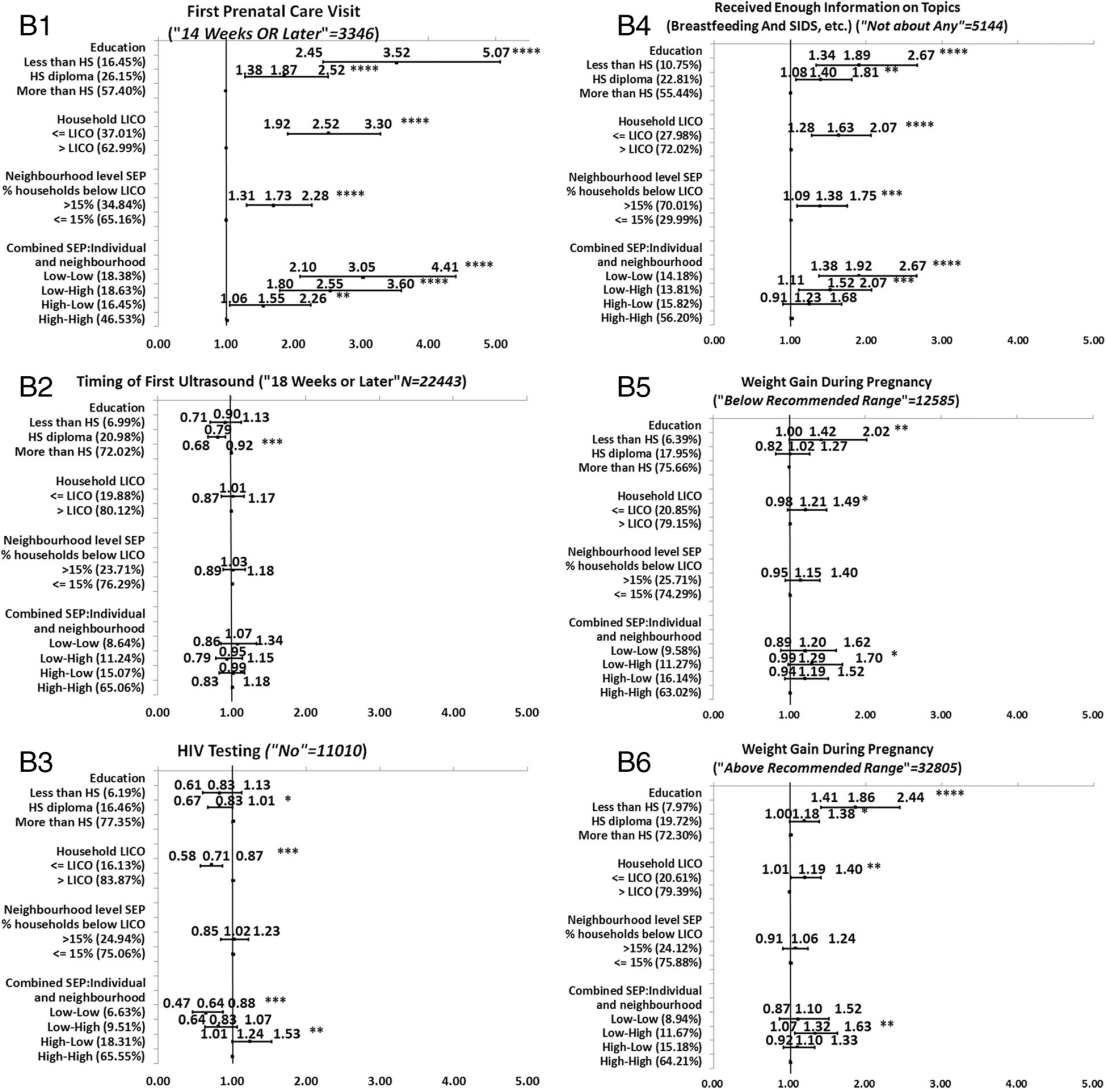
**Associations between individual level SEP (education and household income), neighborhood SEP and combined SEP measures, and the group of prenatal care indicators.**

**Figure 3:**
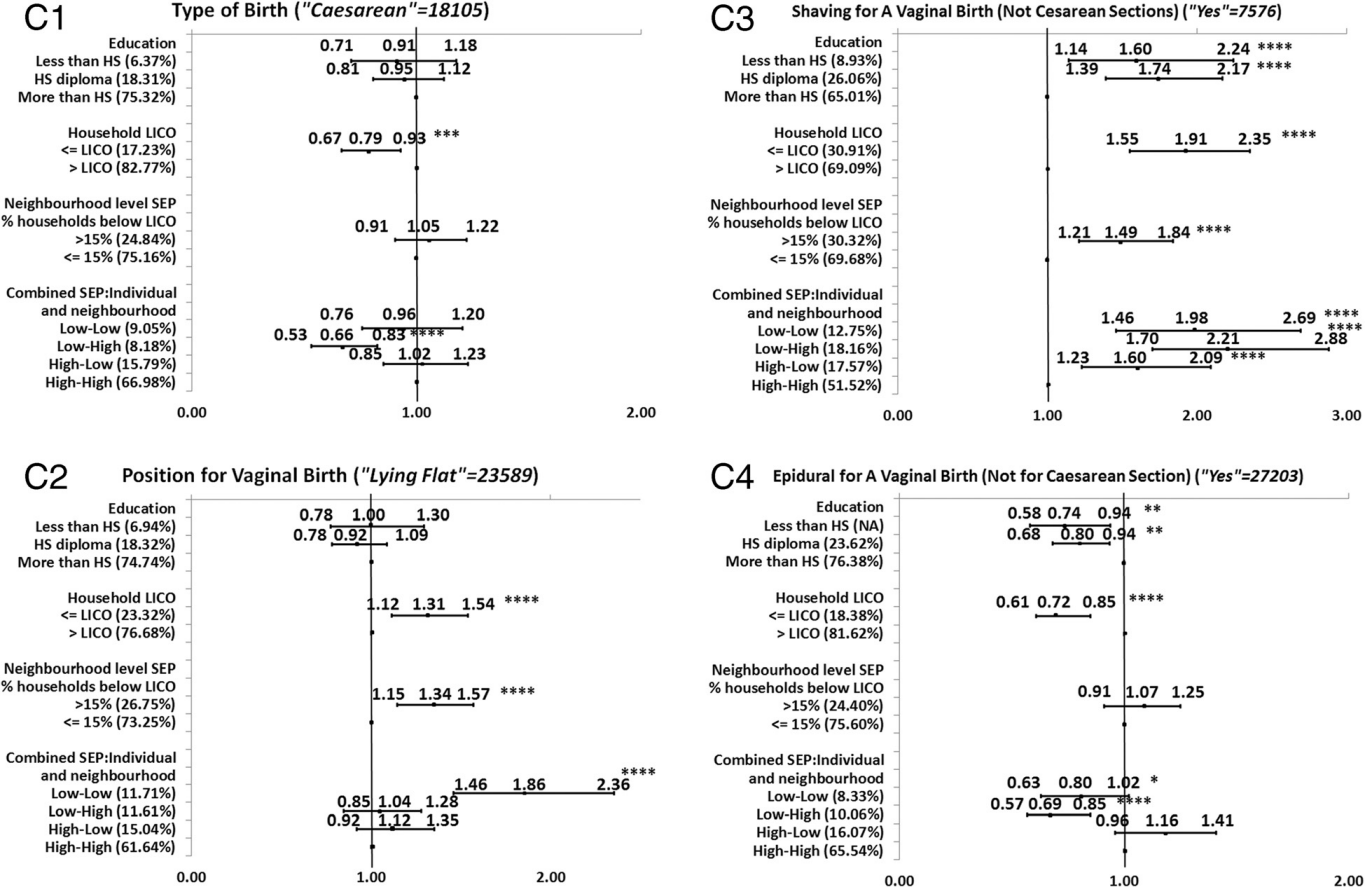
**Associations between individual level SEP (education and household income), neighborhood SEP and combined SEP measures, and the group of labor and delivery experiences.**

**Figure 4:**
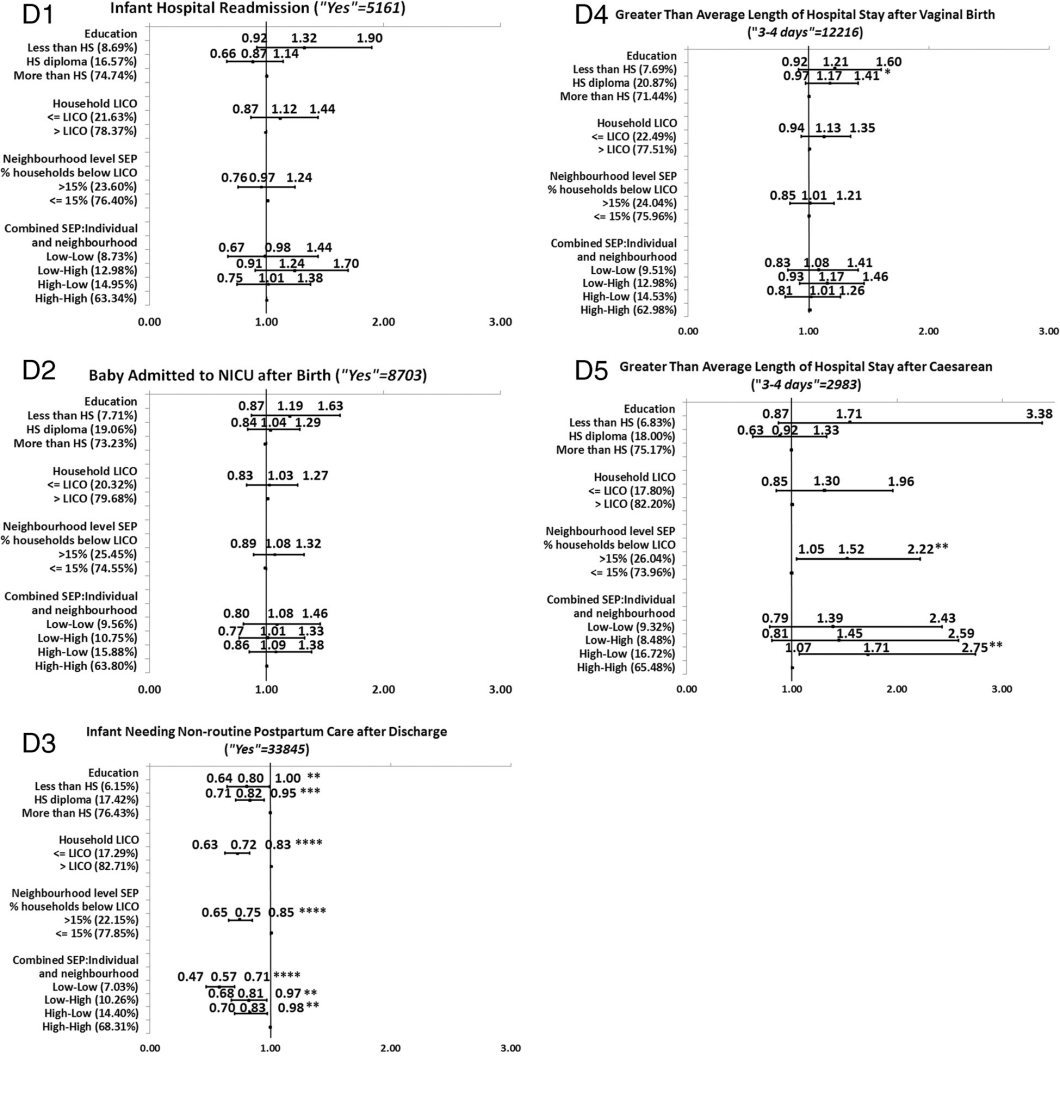
**Associations between individual level SEP (education and household income), neighborhood SEP and combined SEP measures, and the group of indicators of medical care during neonatal period.**

**Figure 5:**
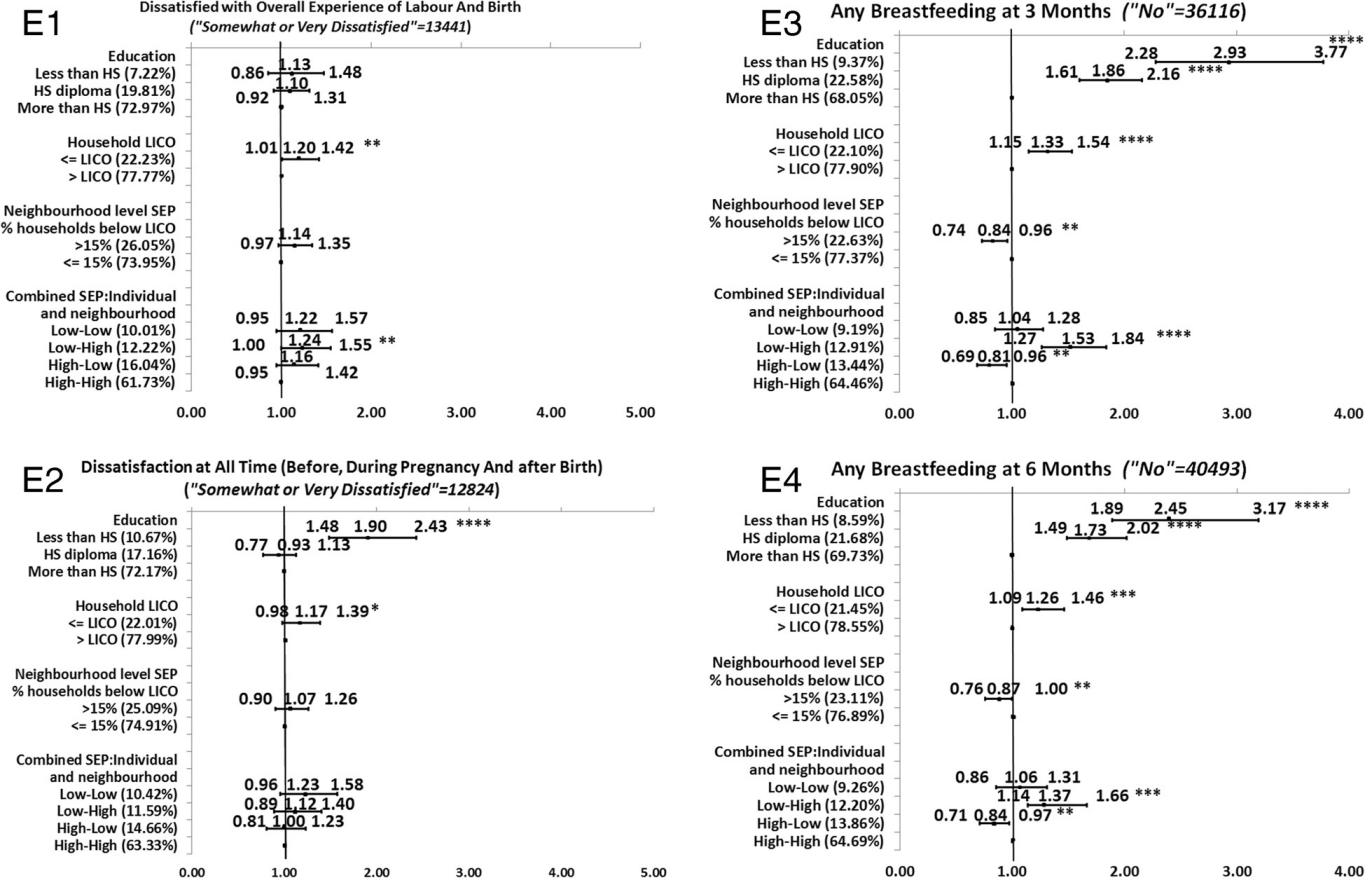
**Associations between individual level SEP (education and household income), neighborhood SEP and combined SEP measures, and the group of post-partum maternal satisfaction and breastfeeding.**

Full description of the level of significance, and the direction and magnitude of the bivariate associations between each of the SEP measures and each MCH outcome is presented in the Figures 1, 2, 3, 4, and 5. We decided to present unadjusted parameter estimates (ORs and 95% CIs) for these associations since adjusting for potential confounders of age and parity in the multivariable models changed the associations with SEP by less than 5% for almost all the MCH outcomes, and, for a few, by just over 5%. We concluded that age and parity were not significant confounders and presented results bivaraite associations. Summary of the findings of the Figures is provided in Table 3. In the following we will detail these findings.

**Table 3:** **Summary of the significance and direction of the associations between SEP measures and MCH outcomes in different groups from the Canadian maternity experiences survey**

	**Education** ^**a**^	**Household income (LICO)** ^**b**^	**Neihgbourhood SEP** ^**c**^	**Combined SEP** ^**d**^
***A- Maternal and infant health status indicators***				
**1. Preterm Birth**	**+**	**NS**	**NS**	+ (*) (low-high)
**2. Small for gestational Age**	**NS**	**+**	**+**	**+** (low-low)
**3. Self rated health**	**+**	**+**	**+**	**+** (low-low)
**4. Postpartum Depression**	**+**	**+**	**+**	**+** (low-low)
**5. Pre-pregnancy BMI** (<18.5)	**+**	**+**	**+**	**+** (low-low)
**6. Pre-pregnancy BMI** (>24.5)	**+**	**+**	**NS**	**+** (low-high)
***B- Prenatal care***				
**7. First prenatal care visit**	**+**	**+**	**+**	**+** (low-low)
**8. Timing of ultrasound**	**-**	**NS**	**NS**	**NS**
**9. HIV testing (no)**	**NS**	**-**	**NS**	**+** (low-low)
**10. Received enough information during pregnancy**	**+**	**+**	**+**	**+** (low-low)
**11. Weight gained during pregnancy (kg) - (Below recommended range)**	**+**	+ (*)	**NS**	+ (*) (low-high)
**12. Weight gained during pregnancy (kg)-more (Above recommended range)**	**+**	**+**	**NS**	**+** (low-high)
***C- Labor and delivery*** **expereinces**				
**13. Type of birth (caesarean)**	**NS**	**-**	**NS**	**+** (low-high)
**14. Epidural for a vaginal birth**	**-**	**-**	**NS**	**+** (low-high)
**15. Shaving for a vaginal birth**	**+**	**+**	**+**	**+** (low-high)
**16. Position for vaginal birth (not laying flat)**	**NS**	**+**	**+**	**+** (low-low)
***D- Medical care during the neonatal period***				
**17. Infant hospital readmission**	**NS**	**NS**	**NS**	**NS**
**18. Infant needing non-routine neonatal care after discharge?**	**-**	**-**	**-**	**-** (low-low)
**19. Baby admitted to NICU after birth**	**NS**	**NS**	**NS**	**NS**
**20. Length of hospital stay after caesarean birth (More than average)**	**NS**	**NS**	+	+ (high-low)
**21. Length of hospital stay after vaginal birth (More than average)**	+ (*)	**NS**	**NS**	**NS**
***E- Postpartum infant care & maternal reflections on the overall experience of health care***				
**22. Any breastfeeding at 3 months**	**+**	**+**	**-**	**+** (low-high)
**23. Any breastfeeding at 6 months**	**+**	**+**	**-**	**+** (low-high)
**24. Overall experience of labor and birth**	**NS**	**+**	**NS**	**+** (low-high)
**25. Satisfaction with previous aspects of maternity care**	**+**	+(*)	**NS**	**NS**

(+) Significant positive association (OR higher than 1) (P < 0.05).

(−) Significant negative association (OR less than 1) (P < 0.05).

(NS) Non-significant association.

(*) Significant at the level of 0.05 < p-value ≤ 0.10.

Reference category: ^a^More than high school, ^b^Above LICO, ^c^More than15% of households below LICO, ^d^High individual-high neighboruhood SEP.

### Direction and consistency of the associations for groups of MCH outcomes

Generally, the five groups of MCH outcomes showed a consistent positive gradient for most (23/25) of the health outcomes across one or more SEP measures; the higher the SEP level, the better the MCH outcome. For the significant associations, mothers with lower SEP (compared to those with higher SEP) had poorer MCH outcomes. Among the five groups of MCH outcomes, we observed more consistent association in Group A of health status indicators of the mother and infant. This was followed by Group B of parental care (Figures 2B1-6), Group C of labor and delivery experiences (Figures 3C1-4), Group E of postpartum infant care and reflection of the overall experience of health care services (Figures 5E1-4), and lastly Group D of medical care during the neonatal period, which had the least significant associations with SEP. Non-significant association was found for infant hospital readmission, admission to the NICU, and greater-than-average length of stay at the hospital (Figures 4D1-5).

While the direction of the significant associations was generally positive (OR > 1), some associations had a negative gradient (OR < 1) (Table 3). That is, those with the highest SEP were more likely to report poorer MCH outcomes than those with the lowest SEP. Those negative gradients were not always in the expected direction. For example, in Group B of prenatal care, there was a negative association between education and timing of the ultrasound. More mothers with higher education conducted the ultrasound later than the recommended time (>18 weeks or later) compared to mothers with lower education (Figure 2B2). Also, mothers with higher household income were more likely to report no HIV testing than mothers with lower household incomes (Figure 2B3). Negative associations were also observed for type of birth (Figure 3C1), and epidural for a vaginal birth (Figure 3C4). In Group D of neonatal care, all SEP measures were negatively associated with infant needing non-routine care (Figure 4D3). Lastly, in Group E of postpartum infant care, neighborhood SEP was significantly and negatively associated with any breastfeeding at 3 or at 6 months (Figure 5E3-E4).

Another important finding is that not all categories of the SEP variables had significant associations with the MCH outcomes. This inconsistency was more evident for the combined SEP measure, which did not show a steep consistent gradient for half (10/20) of the associations with the MCH outcomes. Despite the fact that some CIs overlapped, Table 3 shows that the category low individual-high neighborhood of the combined SEP measure had the highest ORs compared to other categories of this variable.

### The magnitude of inequalities by the SEP measures

Comparing the magnitude of the MCH inequalities (the unadjusted OR (UORs) in the highest and lowest SEP categories, Figures 1, 2, 3, 4, and 5), we found that mothers’ education had the highest ORs and larger inequalities, which was followed by combined SEP, and household LICO. Neighborhood SEP had the smallest magnitudes (point estimates).

For the significant and positive associations, the unadjusted odds ratios (UORs) for education were higher compared to those in other SEP categories. We observed the largest magnitudes between the highest and lowest SEP by education in Group A of health status indicators for the mother and infant (UOR for self-rated health was 3.75, 95% CI: 2.95-5.43) (Figure 1A2), for post-partum depression UOR was 2.44 (95% CI: 1.75-3.41) (Figure 1A4), and for BMI < 18.5, UOR was 3.17 (95% CI: 2.16-4.64) (Figure 1A5). The next strongest association with education came among Group B of prenatal care variables (Figures 2B1-6). For example, the UOR for first prenatal visit was 3.52 (95% CI: 2.45-5.07) (Figures 2B1), and for received enough information UOR was 1.89 (95% CI: 1.34-2.67) (Figure 2B4). The third group in terms of strength of associations with education was Group E postpartum infant care and maternal reflections on the overall experience of health care (Figures 5E1-4). In this group, the UOR of any breastfeeding at 3 months was 2.93 (95% CI: 2.28-3.77) (Figure 5E3). The fourth group in this order was Group C of labor and delivery experiences (Figures 3C1-4), which had only two variables significantly associated with education: shaving for vaginal birth (UOR:1.60; 95%CI:1.14-2.24) (Figure 3C3) and epidural for vaginal birth, however this variable had negative associations with education (UOR: 0.74; 95% CI: 0.58-0.94) (Figure 3C4). Lastly, only one variable out of four in Group D (medical care in the neonatal period) was significantly associated with education but this was a negative association (infant needing non-routine care, UOR:0.80; 95% CI: 0.64-0.99) (Figure 4D3).

Combined SEP was the measure with the second-greatest differences between the highest and a lowest SEP level (Figures 1, 2, 3, 4, and 5). The highest UOR for combined SEP was 3.30 (95% CI: 2.42-4.50) in the association with postpartum depression (Figure 1A4). The patterns of the strength of gradients were similar for combined SEP to what we saw for education; that is, the variables grouped as A, “health status indicators for the mother and infant” had the largest gradients (Figures 1A1-6), while those grouped as B, “medical care during the neonatal period” had the smallest (Figures 4D1-5). However, as we mentioned above, the combined SEP had inconsistent gradients and the category of low individual-high neighborhood showed the poorest health outcomes. Household income was the third-strongest associated SEP measure, with the highest UOR of 2.63 (95% CI: 2.09-3.32) in the association with postpartum depression (Figure 1A4). Household income had the same pattern regarding the associations with the groups of MCH outcomes that we observed with education (Figures 1, 2, 3, 4 and 5).

The smallest magnitudes of inequalities we observed was for neighborhood SEP, with highest UOR of 1.73 (95% CI: 1.31-2.28) associated with the first prenatal care visit (Figure 2B1). Neighborhood SEP was associated with the groups of MCH outcomes in the same trend that we observed for the other SEP measures (Figure 1, 2, 3, 4, and 5). In addition, all health indicators that were associated with neighborhood SEP were also associated with the combined SEP, but the combined SEP showed inequalities for 20 MCH outcomes whereas the neighborhood SEP was only associated with 12 of these health indicators (Figure 1, 2, 3, 4, and 5).

### The magnitude of inequalities within each group of MCH outcomes

Examining the patterns of magnitude of SEP inequalities for the positive associations with UORs > 1 within the groups of outcome variables across the different SEP measures, we found that Group A of health status indicators for mother and infant (Figures 1A1-6) showed the greatest SEP inequalities with an UOR of 3.75 (95% CI: 2.59-5.43) for the association between education and self-rated health (Figure 1A2), and lowest UOR of 1.27 (95% CI: 1.10-1.48) between household income and pre-pregnancy BMI (>24.5) (Figure 1A6). This was followed by the variables in Group B of the prenatal care (Figures 2B1-6) with highest UOR of 3.52 (95% CI: 2.45-5.07) for the association between education and first prenatal visit (Figure 2B1) and lowest UOR of 1.19 (95% CI: 1.01-1.40) for the association between weight gained during pregnancy (above recommended range) and household income (Figure 2B6). Next was Group E of postpartum infant care and maternal reflections on overall experiences of health care (Figures 5E1-4). Highest UOR in this group was 2.93 (95% CI: 2.28-3.77) for the association between education and any breastfeeding at 3 months (Figure 5E3), and the lowest UOR was 1.17 (95% CI: 0.98-1.39) for the association between household income and satisfaction at all times (Figure 5E2). The associations within Group C labor and delivery (Figures 3C1-4) were next, with highest UOR of 1.98 (95% CI: 1.46-2.69) for the association between combined SEP and shaving for vaginal birth (Figure 3C3), and lowest UOR (1.31; 95% CI: 1.12-1.54) in the association between household income and position for the vaginal birth (Figure 3C2). Group D of medical care during the neonatal period had the fewest significant associations and smallest magnitude of effects with SEP measures (Figures 4D1-5). The highest UOR in this group was 1.71 (95% CI: 1.07-2.75) in the association between combined SEP (high individual-low neighborhood category) and greater than average length of stay at the hospital after caesarean section (Figure 4D5), and lowest UOR of 1.17 (95% CI: 0.97-1.41) in the association between education and greater than average length of stay at the hospital after vaginal birth (Figure 4D4).

The magnitude for the negative associations (OR < 1) between SEP measures and MCH outcomes was highest in the association between neighborhood SEP and any breastfeeding at 6 months (UOR: 0.87, 95% CI: 0.76-1.00) (Figure 5E4), and lowest for the association between combined SEP and infant needing non-routine neonatal care (UOR: 0.57, 95% CI: 0.47-0.71) (Figure 4D3).

## Discussion

To our knowledge, this is the first large population-based study to systematically compare the direction and magnitude of individual and neighborhood social inequalities across multiple MCH outcomes categorized into five groups of that represent different needs of mothers and infants in different periods before pregnancy, during pregnancy and after birth. Our findings revealed five key patterns.

### Consistent socioeconomic gradient for most health outcomes, but differing slopes for different groups of outcomes

Overall, we found consistent socioeconomic gradients across most (23/25) of the MCH outcomes in the five groups for one or more SEP measures (individual, neighborhood or both). In general, mothers with lower education, lower household income (at or below the LICO), residing in lower SEP neighborhoods and having low individual-high neighborhood combined SEP had poorer MCH health outcomes than mothers at the higher SEP levels. These results support the already rich literature showing socioeconomic inequalities in MCH outcomes [11,17,18,20,23,42]. However, the current study also identified some groups of MCH indicators where inequalities were steeper than they were in other groups. We found that the SEP measures showed stronger associations (higher ORs) among outcomes belonging to the health status of the mother and infant (Group A), this was followed by prenatal care outcomes (Group B), postpartum infant care and maternal reflection on overall experiences of health care (Group E), labor and delivery (Group C), and, lastly, medical care during the neonatal period (Group D). The last group had the fewest and smallest significant associations with SEP.

The differences in the magnitude of inequalities *between* groups of variables suggest different pathways might be operating between SEP and each of the groups. For example, higher inequalities in Group A of health status of the mother and infant outcomes suggest a cumulative effect, in particular of low SEP, operating prior to and throughout the prenatal period, possibly composed of multiple social determinants of health that affect MCH outcomes and for which SEP is often an indicator (e.g., employment, housing, ethnicity etc.) [18].

### Negative direction for some of the associations

While most of the significant associations between SEP measures and MCH outcomes showed positive gradients; higher SEP measure indicated poorer MCH outcomes, some of the associations with MCH outcomes were negative where women with higher SEP were more likely to experience the poorer outcome). These negative associations were more prevalent during birth and delivery (Group C) and medical care during the neonatal (Group D), for example higher-income women being less likely to have prenatal HIV testing and more likely to conduct a late first ultrasound. Reasons for these observed associations are not clear and should be the subject of future investigations. Our finding support the growing literature showing that receipt of epidural for vaginal birth is more likely among women of higher SEP [43]. However, our finding that caesarean sections occurred more frequently among those with higher income has not been reported in previous Canadian studies. One study showed that age-adjusted caesarean sections were higher in women from low-income neighborhoods [44]. However, another study from Nova Scotia, Canada, showed that neonatal medical interventions, such as induction of labor and caesarean section, did not differ by income [45]. One explanation for these differences is that obstetric practices may vary by province and that Nova Scotia differs from other provinces included in our study. Alternatively, obstetric practices may have changed since the time of the Joseph et al. study [46-48]. Results of past studies, such as Joseph et al., might be related to the universal access to these services in Canada as national public health care systems have been shown to increase equity of access to health care and result in fewer health inequalities in multicounty comparisons [46-48]. Thus, if funds and services were to be cut, we might see a change in the relationship between medical care and SEP similar to the changes observed in Australia where there was an increase in caesarean sections as a result of a shift from public towards private hospitals for delivery [49,50].

### Steeper gradients for maternal education compared to other SEP measures

Maternal education had steeper gradients in the associations with different MCH outcomes (UOR up to 3.75, 95% CI: 2.59-3.75 in the association with self-rated health), followed by combined SEP (UOR up to 3.30, 95% CI: 2.42-4.50 in the association with postpartum depression), then household income (UOR up to 2.63, 95% CI: 2.09-3.32 in the associations with postpartum depression), and, finally, neighborhood income (UOR up to 1.73, 95% CI: 1.31-2.28 in the association with first prenatal visit). However, household income and combined SEP revealed more frequent statistically significant associations than those observed for maternal education. This result corresponds with a systematic review which indicated that income was more frequently associated with birth outcomes than education [18]. The results also suggests that education is different from income and combined SEP and should not be used interchangeably as a proxy for those measures [10,20].

The pattern we found, in which maternal education had a steeper gradient between high and low SEP than did the other measures, indicates that maternal education can reveal the depth of health disparities regarding MCH indicators. Previous studies have shown that maternal education is associated with many MCH outcomes, including preterm birth and stillbirth [14,15], low birth weight [51], inadequate use of prenatal healthcare services [17], and risky maternal behaviors during pregnancy [52]. Lower education, meanwhile, has been associated with preterm birth, small for gestational age, and other complications [11]. There may be multiple pathways linking education and maternal and infant health status outcomes. Maternal education might reflect not just individual and household economies, but other pathways, such as access to information and knowledge, problem solving, social networks and involvement, and social prestige [4].

### Higher magnitude of MCH inequalities for individual-level SEP than for neighborhood-level SEP

In our study, neighborhood SEP alone exhibited the weakest magnitude of the associations with MCH outcomes. This might have been expected, as prior studies have also found significant but small effects of neighborhood measures on a variety of MCH outcomes [23,27-29,31]. Despite increased attention on the effects of the social environment on health, few studies were conducted on neighborhood SEP compared to individual-level SEP [4]. Also, our finding that MCH outcomes are more strongly associated with individual-level SEP than with neighborhood SEP might reflect the fact that neighborhood SEP in our study was an aggregate of household LICO, whereas household LICO itself might be a more accurate and direct reflection of resources available to a household. Studies using median income to measure neighborhood SEP (as opposed to neighborhood-derived variables based on education or employment) have shown consistent associations with increased risk of preterm birth [13,28].

### Steeper gradients for combined SEP compared to household income

Combined SEP showed significant associations, which were similar to those found for household income; however, the magnitude of the associations was higher for the combined SEP. Combined SEP might be a more powerful measure than individual income alone or neighborhood SEP alone in revealing social inequalities in MCH since it takes into account both the individual and neighborhood dimensions of SEP. In addition to individual achievement, it captures resources or problems at the neighborhood level (e.g., high-income neighborhoods may have more facilities and services, fewer neighborhood problems and lower crime rates that reflect larger socioeconomic and social processes and policies) [25]. For example, life-long residency in a low-income neighborhood has been associated with low birth weight, and this association was stronger among women from ethnically identified groups [53].

Most of our outcomes (23/25) were associated with combined SEP. The magnitude of the associations with combined SEP was weaker than it was for education, but stronger than that for household income and neighborhood SEP. The consistency of associations for the combined SEP variable in the current study indicates the added value for measuring multiple levels of SEP simultaneously. The combined SEP was constructed based on aggregate income in a neighborhood [39]. While individual SEP and neighborhood SEP represent different sources contributing to socioeconomic inequalities, the combined SEP measure disenables the cumulative effects of individual and contextual exposures. For example, combined SEP might capture specific mediators and pathways acting between residential neighborhoods SEP and mothers’ education or income, such as social networks, social cohesion, and/or higher use of health care services among mothers with higher SEP [18]. This allows us to consider how studies that use one level of SEP might either show higher estimation than the actual effect of a SEP measure, or over-adjust for the SEP effect when adjusting for many individual SEP measures.

However, the combined SEP did not show a consistent gradient for all associations. In 10/20 of the significant associations, the category of low individual-high neighborhood SEP showed the greatest difference from the reference category of high individual-high neighborhood SEP. This suggests that the financial and psychological strain of being low income within a high-income neighborhood contributes to adverse health [54,55] and should be the focus of futures studies to unpack the pathways by which individual and neighborhood SEP simultaneously impact health.

#### Strength and limitations

This study has several limitations. First, because of its cross-sectional design, causal inferences cannot be drawn from our results. Second, our sample included only mothers whose singleton birth babies were living with them at the time of the interview (on average 9 months after birth), which affects the representativeness of the sample. This means that mothers with multiple births or mothers whose babies died or were separated from them prior to the interview were excluded. Third, we did not stratify by ethnicity, immigrant or Aboriginal status. Previous studies have shown that ethnicity and racialization are related to systematic, structural and/or institutional discrimination that intensify social vulnerability and worsen health [23,42,53,56]. Fourth, odds ratios may not have been the ideal measure of association for outcomes that have a prevalence greater than 10% [57].

The strength of our data is that they are drawn from a nation-level survey. The data also examine groups of MCH outcomes that represent mother’s and infant’s health care needs during three periods: before pregnancy, during pregnancy and after birth; examining SEP inequalities in health across these periods, and for groups of outcomes, facilitates an understanding beyond what we typically get in studies that examine single outcomes or single SEP indicators [58]. While many studies present adjusted data, which might attenuate SEP inequalities [18], in this study our focus is specifically on the effects of SEP on MCH outcomes and our findings were found not to be confounded by age and parity.

## Conclusions

Our findings on the patterns (magnitude and direction) of differences across a wide range of MCH outcomes revealed consistent patterns of health inequalities, with deeper inequalities for the outcomes grouped under maternal and infant health status. The stronger gradients in this group of outcomes suggest a larger role for the social determinants of health than those grouped under prenatal care, birth and delivery and postpartum infant care or maternal perceptions of care. Medical care during the neonatal period had the fewest associations with SEP suggesting a smaller role for the social determinants of health for this group of outcomes.

With regard to differences by measure of SEP, the magnitude of MCH inequalities was higher when individual-level SEP was used than when we considered neighborhood SEP. In particular, education showed the greatest gradients compared to household income, combined SEP, and neighborhood SEP. Combined SEP helped to disentangle the joint influence of individual and neighborhood level SEP often showing that individuals in the category of low individual-high neighborhood SEP has the poorest health outcomes compared to the other three categories in this measure. Taken together, our findings suggest hypotheses for future research on SEP inequalities, which might inform policies to address the longstanding gradients.

## Abbreviations

SEP: Socioeconomic position
MES: Maternity experiences survey
MCH: Maternal and child health
OR: Odds ratio
UOR: Unadjusted odds ratio
LICO: Low income cut-off point
BMI: Body mass index
NICU: Neonatal Intensive Care Unit
